# Picrosirius‐Polarization Method for Collagen Fiber Detection in Tendons: A Mini‐Review

**DOI:** 10.1111/os.12627

**Published:** 2021-03-10

**Authors:** Jie Liu, Ming‐you Xu, Jing Wu, Hao Zhang, Li Yang, Deng‐xing Lun, Yong‐cheng Hu, Bin Liu

**Affiliations:** ^1^ Tianjin Medical University Tianjin China; ^2^ Center for Medical Device Evaluation NMPA Beijing China; ^3^ Tianjin Hospital Tianjin China

**Keywords:** Birefringence, Collagen, Morphology, Picrosirius red staining, Polarized light microscopy

## Abstract

Although the structure and composition of collagen have been studied by polarized light microscopy since the early 19th century, many studies and reviews have paid little or no attention to the morphological problems of histopathological diagnosis. The morphology of collagen fibers is critical in guiding mechanical and biological properties in both normal and pathological tendons. Highlighting the organization and spatial distribution of tendon‐containing collagen fibers can be very useful for visualizing a tendon's ultrastructure, biochemical and indirect mechanical properties, which benefits other researchers and clinicians. Picrosirius red (PSR) staining, relying on the birefringence of collagen fibers, is one of the best understood histochemical methods that can highly and specifically underline fibers better than other common staining techniques when combined with polarized light microscopy (PLM). Polarized light microscopy provides complementary information about collagen fibers, such as orientation, type and spatial distribution, which is important for a comprehensive assessment of collagen alteration in a tendon. Here, this brief review serves as a simplistic and important primer to research developments in which differential staining of collagen types by the Picrosirius‐polarization method is increasing in diverse studies of the medical field, mainly in the assessment of the morphology, spatial distribution, and content of collagen in tendons.

## Introduction

A tendon is most often thought of as a bright white connective tissue, which primarily contains dense longitudinal fibrils (10–350 nm in diameter) that braid spatially into rope‐like collagen fibers (1–20 μm in diameter), transferring tensile loads from muscle to bone to facilitate joint motions^1^, ^2^. The distinct arrangement of collagen fibers, together with their interactions with the noncollagenous matrix rich in hydrophilic proteoglycans, endows a normal tendon with unique mechanical functions, such as resistance to tension and the transmission of forces^3^, ^4^. Moreover, the degradation and loss of collagen fibers are generally associated with functional decline^5^, ^6^. Accordingly, understanding the architecture and biomechanics of collagen fibers is fundamental to exploring how compositional differences initiate altered biological and mechanical behaviors of a tendon^7^.

The distribution and quantitative estimation of collagen fibers can be obtained by many microscopic techniques and morphological methods applied to sections with different degrees of satisfaction. Among these methods, light microscopy, which is commonly used in histology, does not provide sufficient imaging resolution to reveal the detailed architecture of tendons at the ultrastructural level but shows the general morphology of tenocytes and the tendon texture^8^. Transmission and scanning electron microscopy can provide high‐resolution images of well‐defined individual collagen fibrils but these are relatively expensive, involve complicated and time‐consuming specimen preparation, and provide nanolevel details that are redundant for many studies^9^. Alternative optical techniques are thus needed to better visualize collagen fibers in both physiological and pathological tissues.

Furthermore, among the various histological methods, immunohistochemistry allows a detailed phenotypic analysis of specific collagen fibers and their spatial distribution patterns^10^. However, it is affected by several limitations and pitfalls, such as complex and time‐consuming protocols and expensive reagents, in contrast to histochemistry. Moreover, collagen staining patterns exhibit marked variability in assays for different commercially available antibodies^11^. Compared to them, histochemistry represents a simple and quick method to detect the total collagen fiber content in tendon sections. In this regard, the majority of stains that are currently used are traditional trichrome stains (e.g., the Masson, Mallory, and van Gieson methods). Further studies, however, have found that these methods underestimate the collagen fiber content in certain circumstances^7^, ^12^. One explanation for this funding is that these techniques lack selective precision, failing to detect the very thin collagen fibers observed in electron microscopy. Except for these confounding issues, the tendency of a stain to fade prompted Puchtler *et al*.^12^ to seek a better assessment method for collagen alternation, that is, Picrosirius red staining.

Picrosirius red staining (also called PSR or “Sirius red” staining) is a selective histochemical method suitable for both morphological and semiquantitative detection in paraffin‐embedded sections of both normal and pathological tendons. In sections that are analyzed with this procedure under polarized light microscopy (PLM), a complex mixture of different colors, representing various collagen types, is simultaneously presented, which is useful for identifying and subsequently analyzing the differential distribution patterns of the structurally distinct collagen types in a tendon^13^, ^14^. Furthermore, PLM can be performed with a light microscope by adding two filters, a polarizer and an analyzer, and is a powerful and robust tool for the assessment of fiber properties in tissues containing collagen fibers^15^. Jungueira *et al*.^16^ first described a method for the detecting and subsequent analysis of collagen fibers stained using a combination of Sirius red staining and polarized light microscopy. Currently, with image analysis technologies, this method exploits elaborate data processing and polarized light imaging and continues to be widely used for the identification and analysis of the fiber anisotropy, orientation^17^, ^18^, and crimp pattern^19^ in animals^20^ and humans. In this mini‐review, we discuss the basic concepts of tendon morphology related to polarization microscopy; readers who are interested in a more in‐depth discussion of this topic can consult many excellent studies and reviews^1^, ^5^, ^7^, ^21^, ^22^.

The Picrosirius‐polarization method for collagen: fundamental knowledge.

Anisotropic objects can exhibit a variety of phenomena, including birefringence, which is mainly emphasized in previous studies. In the phenomenon of birefringence, as light enters anisotropic compounds or structures, the light beam is split into two beams with different velocities whose vibration planes are perpendicular to each other^23^. Birefringence is a measure of the difference in the refractive indices of two separate components (the fast ray and slow ray) of the incident ray passing through a sample^24^. Examples of tissues with birefringent constructs include tendons, dermis, muscle, cartilage, and so on.

The presence of anisotropy is attributed to pressure, stretching or directional movement, which usually triggers a remarkably regular pattern of collagen molecules and, consequently, birefringence. In contrast, factors causing a random alignment of molecules, such as healing, usually induce an absence of anisotropy^21^. Interestingly, only the birefringence of longitudinally cut collagen fibers is evaluated, because the longitudinally cut fibers are anisotropic while cross‐sectioned fibers are isotropic^12^. This phenomenon was also discussed in research by Rieppo *et al*.^25^. Furthermore, polarized light imaging (linear, elliptical, and circular) was utilized to visualize the birefringence of collagen fibers stained by Sirius red revealing changes in the hue, alignment, and distribution patterns in sections. In addition, this technique can measure the relative phase change between two polarized lights, which is termed retardance, to quantify the birefringent property of a tendon. When rotating the sections, destructive interference between the ordinary and extraordinary rays emitted from a biological sample can be observed through the ocular lens of PLM, which is characterized by a dark background, and the angle of the microscope is recorded. Subsequently, the optical retardation of the sample is obtained by the calculation formulas mentioned in previous literature^26^. The relative magnitude of the retardance is positively correlated with the density, alignment, and thickness of biological samples^27^.

Picrosirius red dyes are elongated birefringent molecules, and when bound within the upper groove of well‐oriented collagen molecules that are eosinophilic, the dyes orient parallel to the long axis of each collagen fiber, thereby greatly enhancing the normal birefringence stemming from a uniform fiber distribution^21^, ^28^. Undoubtedly, collagen fibers stained with Sirius red can be detected with polarized light, where type I collagen fibers appear bright and red‐yellow, in sharp contrast to type III collagen which appears green^16^, ^24^. Therefore, this method is widely employed for quantitative estimations of tissues, because it enables morphology identification and a better quantitative assessment than the van Gieson method^7^. In addition, morphometric image analysis has been widely applied to assess the properties of collagen fibers due to its precision, objectivity, and reproducibility^29^, ^30^. Therefore, PSR staining can be employed with less fading than the van Gieson or other methods and subsequently visualized with much better results with the aid of PLM in combination with morphometric image analysis due to the varying birefringent properties of cationic collagen fibers combined with the elongated, anionic Sirius red dye^12^, ^31^.

### Assessment of the Collagen Fiber Morphology

**Fig 1:**
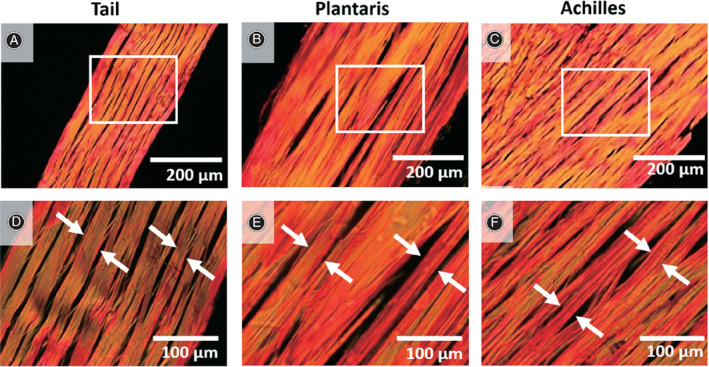
Assessment of collagen fiber structure in longitudinal direction. (A‐C) Rat tail, plantaris, and Achilles tendons were cut in longitudinal direction, stained with picrosirius red, and showed at low magnification. White boxes represent areas observed at (D‐F) high magnifications, and collagen fibers are highlighted (white arrows). Adapted with permission from Lee *et al*.^33^.

Photographs and descriptions of the patterns of various tendon tissues have been presented previously. Most of the literature has focused on directly observing and analyzing the differences in the hue, alignment, and distribution patterns of collagen fibers in tendon sections. For instance, according to the description by Jiang *et al*.^20^, when using polarized light microscopy large amounts of orientationally ordered type I collagen were clearly visualized using the special stain (PSR) in healthy, asymptomatic Achilles tendons, which appear as thick, brilliant (strongly birefringent) red‐yellow fibers resulting from the interaction of the molecules of type I collagen with the Sirius red dye; in contrast, type III collagen is weakly birefringent and characterized by thin greenish fibers. Cury *et al*.^32^ reported similar colored patterns when studying the changes in collagen fiber types of adult and elderly rat calcaneal tendons. Moreover, in 2019, Lee *et al*.^33^ utilized a variety of microscopes, such as polarized light microscope (Fig. 1), electron microscope and second harmonic microscope, to characterize the structure of collagen in rat tendons.

Moreover, various research groups have found that a few weakly birefringent collagen fibers observed with PLM are explained by immature type III collagen fibers that arranged in a disorderly manner, forming a network structure and causing a decrease in their anisotropy^12^. Supporting these notions, Brown *et al*.^34^ found that the Picrosirius‐polarization method allows one to highlight type I collagen, which appears as a well‐defined, red or yellow fibrillary element that stands out from the green‐stained type III collagen fibers scattered around type I collagen in a sparse network. In 2019, Hutson *et al*.^35^ investigated and subsequently compared the microstructure of collagen fibers in rat tails that had been fixed in formalin, flash frozen, or preserved in an ammonium sulfate solution. The authors used Picrosirius red staining to observe different colors and the distribution pattern; more importantly, they quantified and analyzed the area percentage of total tissue regions that exhibit birefringence and found that the three preservation methods had little impact on the collagen fiber measurements. However, under pathological conditions in which collagen degradation, consequent molecular disorder and irregular arrangement, and content change occur, the birefringence will exist in a different pattern than that in normal tissue^36^. The results of these studies have deepened scholars' understanding of the possibilities and limitations of the Picrosirius‐polarization method in analyzing collagen fibers.

Furthermore, many scholars have also proposed and used other microscopic techniques to investigate the impact of different treatments or pathological conditions on collagen fibers. For example, Yildirim *et al*.^37^ utilized second‐harmonic microscopy to distinguish normal and scarred tissues by determining the orientation and density of collagen fibers in the image. Oliveira *et al*.^38^ revealed the morphology of the collagen fibers in the Achilles tendons of diabetic rats using atomic force microscopy. The authors found that the tendons of diabetic rats showed pathological changes, such as thickening of the tendon, disordered arrangement of the fibers, and diminished biomechanical properties, compared with healthy tendons. Additionally, Zabrzyński *et al*.^39^ presented electron microscopy images of collagen fibers, revealing that the collagen fibrils in pathological tendons are more loosely and disorderly arranged than normal tendons, with fewer coarse collagen fibers and more fine collagen molecules.

Notably, in numerous studies, scholars did not use the microscopy alone to observe the collagen fibers in the tendon but combined the microscopy with other technologies to better observe and analyze the collagen. For instance, Wegner *et al*.^40^ extended the versatility of PSR by using fluorescent imaging to detect the Sirius red signal and applying morphometric image analysis. The authors compared this method with polarization microscopy for collagen imaging and noted that more stained collagen molecules were detected by fluorescent microscopy than by polarized light imaging. Although the collagen fibers appeared as a single color under fluorescent light, there was no apparent shift in the collagen fiber color or density as the sample was rotated. Additionally, to better visualize and describe the complicated three‐dimensional collagen fiber architectural features of the eye, in 2018 Yang *et al*.^18^ proposed and validated 3dPLM as an imaging technique based on PLM that allows for the visualization and further quantification of the collagen fiber orientation in three‐dimensional ocular tissues; in turn, this enables an observation of the tendon's architecture and enriched knowledge of the role of collagen fiber biomechanics in both normal and pathological tissues.

**Fig 2:**
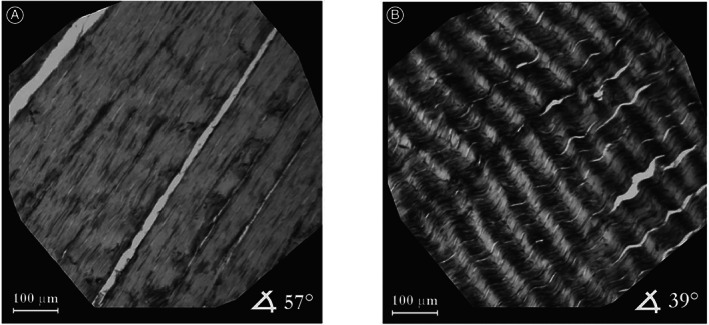
Typical spatial distribution of local orientations of collagen fibers in Achilles tendons, resolution 1 × 1 pixel. Image (A) shows the area where nearly all collagen fiber bundles are aligned at 57°. In contrast, image (B) depicts a specimen of Achilles tendon containing undulated collagen fibers. Adapted with permission from Novak *et al*.^43^.

Another consideration is the crimping phenomenon (length and angle of the crimp peaks). The crimp period, showing alternating dark and light transverse bands, is determined by calculating the length of a line (parallel to the longitudinal axis of the tendon) connecting the apex of adjacent peaks^4^, ^41^. Using polarized light microscopy alone, Mazon *et al*.^42^ noted that the calcaneal tendon of rats has a characteristic crimp pattern with a uniform periodic visible pattern, which is similar to the previous findings by Novak *et al*.^43^(Fig. 2). Moreover, the presence of a crimp period in paraffin‐embedded tissue sections was also confirmed by using hematoxylin/eosin staining, analyzing the tendon sections by light microscopy^6^, ^44^, and using van Gieson staining^45^. Furthermore, Franchi *et al*.^46^ described the structural modifications of collagen fibers in crimps of both relaxed and stretched rat Achilles tendons using polarized light microscopy. The authors found that when a rat Achilles tendon is physiologically relaxed *in vivo*, the tendon crimps increase in number (39.6 in relaxed tendons *vs* 74.4 in stretched tendons, *P* < 0.01) and show more undulation with a decrease in the crimp top angle (148° in relaxed tendons *vs* 165° in stretched tendons, *P* < 0.005). According to the current interpretation, the crimp can indicate the actual functional condition and act as a shock absorber during physiological stretching of the tendon^47^.

### Evaluation of the Collagen Fiber Content and Birefringence

In addition to evaluating the hue of fibers and the spatial distribution of the various colors, combined with image‐analysis software, the Picrosirius‐polarization method has a key advantage over traditional techniques in both identifying fibers and subsequently quantifying the collagen content and birefringence, such as the proportions of different colored fibers and retardance^7^. However, there is very little literature reporting on the measurement of the collagen fiber content of the tendon using polarized light microscopy. Terena *et al*.^48^ described the effect of low‐level laser therapy on the collagen fiber content in tendons during compensatory overload of the plantar muscle in rats. After color separation of the images using the Image J program, the authors found that the irradiated hypertrophy group had a greater proportion of type I collagen in the tendons in comparison to the hypertrophy group after 7 and 14 days. Besides, Zerbinati *et al*.^49^ observed the effect of subcutaneous injection of calcium hydroxylapatite (CaHA) on the collagen fibers of the skin. Under polarization, the authors found that the skin of the experimental group exhibited decreased type I collagen content (40.59% in the control group *vs* 15.54% in CaHA injection group, *P* < 0.01) and an elevated immature type III collagen level (1.76% in the control group *vs* 34.42% in CaHA injection group, *P* < 0.01).

Undoubtedly, the spatial distribution pattern of different collagen fibers stained by Sirius red, from weakly anisotropic to strongly anisotropic staining, can be clearly visualized using a polarizing microscope with image analysis technologies^48^. While the thicker type I collagen fibers appear red/yellow in color, the thinner mature type III collagen fibers have a green color. This differential staining permits a quantitative assessment of types I and III collagen by calculating the ratio of different color areas. In previous research by Alves *et al*.^50^, the collagen proportional area was also utilized in transmission electron microscopy to observe the changes in the microarchitecture of collagen fibers in sections of a reconstructed anterior cruciate ligament with a hamstring autograft *in vivo*. Additionally, to better visualize and analyze the different structural appearances of collagen fibers in horse tendons, in 2012, Södersten *et al*.^51^ employed an immunohistochemistry method and found that more intense immunoreactivity for type III collagen was observed in injured tissues and the disorganized regions than in normal tissue. Recently, in 2019, Woessner *et al*.^52^ utilized quantitative polarized light imaging (QPLI) to investigate and analyze the differences in the collagen fiber content and alignment of mouse skin wounds. QPLI‐derived maps of the collagen fiber organization illustrated the alignment of the fibers, average light retardation (thickness of the collagen fiber layer), and changes in the collagen‐positive pixel density (collagen content) over time. The authors found that the collagen fiber content and layer thickness increased with time in the wound bed. Moreover, a significant increase in the fiber directional variance from day 5 to 10 (*P* = 0.003) indicated that more locally formed fibers were randomly distributed.

In principle, immature collagen fibers are scattered and disordered under the microscope, and highly oriented collagen fibers develop gradually during the maturation process^12^. The above findings aligned with the research of Kim *et al*.^53^, and the authors noted that type I collagen fibers are closely related to mechanical strength, with a relatively larger average fiber diameter, while type III collagen fibers are involved in tendon injury repair. Additionally, the data of Provenzano *et al*.^54^ indicated that the production of type III collagen fibers slowly decreases during development and then increases by a considerable amount after injury in the healing process.

In addition to the collagen proportional area analyzed by morphology in morphometric image analysis, in recent years, some scholars have also proposed and adopted complicated formulas to quantify and analyze the birefringence of biological samples using retardation, which is not commonly discussed in previous literature^4^, ^26^, ^55^. Notably, the measurement of birefringence using crossed light in biological samples requires the tendon to be unstained. Several years ago, Vidal *et al*.^56^ demonstrated that the optical retardation of collagen after Picrosirius staining increased by five to six times. This result can explain why most scholars have tended to stain collagen fibers when characterizing collagen fibers with a polarizing microscope. In fact, the relative magnitude of the retardation is correlated with the density, orderly alignment, and thickness of the samples. In 2018, Spiesz *et al*.^4^ compared the birefringence of the two main components of equine tendons and found that fascicles have a higher retardation than the interfascicular matrix. Therefore, the authors demonstrated that fascicles in the tendon contain a higher density of collagen fibers than the interfascicular matrix. Furthermore, in research by Silva *et al*.^26^ using rat models, the experimental group was treated with a low‐power red laser parallel to the long axis of the tendon. As indicated by their calculations and analysis of the birefringence of nonirradiated and irradiated collagen in unstained paraffin sections, the authors found that the irradiated samples presented significantly higher retardation values than the control samples, regardless of whether the samples were immersed in water or glycerin. Therefore, the authors demonstrated that a low‐power red laser parallel to the long axis of the tendon promotes further arrangement of the collagen fibers in a healthy tendon. This conclusion is consistent with the conclusion described by Terena *et al*.^48^.

### Summary

In conclusion, PLM in combination with Sirius red staining is an inexpensive, simple, and reliable assessment method that can be used to reveal the presence of the collagen fibers, describe their distribution patterns, and perform quantitative detection of the fibers. Indeed, this method allows one to clearly visualize well‐defined type I collagen, which stands out from green‐stained type III collagen, and to obtain limited quantitative results by image analysis in paraffin‐embedded sections of the tendon, under both normal and pathological conditions. However, there are still many confounding problems that needed to be elucidated. On one hand, most experimental studies are limited to smaller animals (e.g., rats and rabbits), and there are few corresponding clinical studies. On the other hand, these technologies, which focus only on two‐dimensional measurements, have a low measurement accuracy and provide limited information about the complex three‐dimensional orientation of collagen fibers. Nevertheless, with the emergence of staining, microscopy, and image analysis technologies and scientific concepts in multiple disciplines and fields, the architecture and composition of tendons will be further studied.
